# Comparison of three *Coxiella burnetii* infectious routes in mice

**DOI:** 10.1080/21505594.2021.1980179

**Published:** 2021-09-27

**Authors:** Halie K. Miller, Rachael A. Priestley, Gilbert J. Kersh

**Affiliations:** Rickettsial Zoonoses Branch, Centers for Disease Control and Prevention, Atlanta, Georgia

**Keywords:** Q fever, aerosol, intraperitoneal injection, oral gavage, ingestion, inhalation, zoonoses

## Abstract

Evidence suggests that *Coxiella burnetii*, which is shed in the milk, urine, feces, and birth products of infected domestic ruminants, can lead to Q fever disease following consumption of unpasteurized dairy products; however, *C. burnetii* is not believed to be a major gastrointestinal pathogen. Most infections are associated with inhalation of aerosols generated from the excreta of domestic ruminants. We recently demonstrated that *C. burnetii* delivered by oral gavage (OG) resulted in dissemination and an immune response; however, it is unclear how infection via the oral route compares to other well-established routes. Therefore, we delivered three strains of *C. burnetii* (representing three pertinent sequence types in the United States, such as ST16, ST20, and ST8) to immunocompetent mice in four doses via aerosol challenge (AC), intraperitoneal injection (IP), or OG. Low dose (10^5) of ST16 by OG was insufficient to cause infection, yet doses 1,000- or 100-fold lower by IP or AC, respectively, induced a robust immune response and dissemination. Despite being able to induce an immune response in a dose-dependent manner, administration of *C. burnetii* via OG is the least efficient route tested. Not only were the immune responses and bacterial loads diminished in mice exposed by OG relative to AC or IP, the efficiency of transmission was also inferior. High doses (10^8) were not sufficient to ensure transmission to 100% of the ST20 or ST8 cohorts. These results may provide some basis for why ingestion of *C. burnetii* as a mode of Q fever transmission is not often reported.

## Introduction

There are five main routes of transmission that bacteria utilize to gain entry into the human host including direct contact, fomites, aerosol, oral, and vector-borne [1]. The causative agent of Q fever, *Coxiella burnetii*, is a bacterial pathogen suspected of employing each of these routes, although infections are most commonly associated with inhalation of aerosols generated from the excreta of domestic ruminants [2]. Upon entry to the human host, *C. burnetii* replicates inside a parasitophorous vacuole in a wide range of cell types with monocytes and macrophages as the primary targets [3]. Q fever disease can occur as an acute infection which may be asymptomatic; however, when symptoms are present, they often manifest as a febrile illness and can include chills, malaise, and myalgia [4]. While acute Q fever is typically self-limiting with a low mortality rate, the disease can recrudesce months to years later, resulting in a serious chronic infection. Chronic Q fever most often presents as endocarditis or vascular infection, which can be fatal without treatment [4].

Transmission of *C. burnetii* by the aerosol route was never more apparent than in the unprecedented Netherlands outbreak between 2007 and 2010, where more than 4,000 confirmed cases occurred in areas surrounding infected dairy goat farms [5]. In 1989, a large outbreak of Q fever occurred in Birmingham UK in a residential area 11 miles from the infected farms [6]. Even though viable *C. burnetii* can be shed in the milk of infected domestic ruminants and evidence suggests that Q fever can occur through consumption of unpasteurized milk, *C. burnetii* is not believed to be a major gastrointestinal pathogen [7–10]. Identifying the source of *C. burnetii* infection can prove challenging when *C. burnetii*-infected milk and milk-based products have been consumed as contamination of the airway may also occur. This is further complicated by the fact that aerosolized *C. burnetii* can travel such long distances, and persons who consume raw milk may be more inclined to visit or live near farms. This ambiguity was the focus of a recent study, where we demonstrated that *C. burnetii* delivered directly into the stomachs of immunocompetent mice led to colonization of gastrointestinal tissues, dissemination, and an immune response [11]. Additionally, we and others have found that mice infected via the oral route display decreased pathology relative to mice receiving the same dose via intraperitoneal injection [12]. Although we have confirmed that oral infection by *C. burnetii* is possible in mice, studies such as the one conducted by Krumbiegel *et al*. demonstrated that of 37 volunteers who consumed raw milk naturally infected with *C. burnetii* none developed any evidence of infection [13]. To begin to unravel this discrepancy, we sought to determine whether infection via the oral route is dose dependent and evaluate how ingestion compares to inhalation. Improved knowledge of these routes of infection will lead to improved prevention and control strategies for Q fever; therefore, we sought to determine how infection via the oral route relates to the well-established routes, inhalation, and injection. To this end, we delivered three strains of *C. burnetii* to mice in four doses via three routes of infection, aerosol challenge (AC), oral gavage (OG), and intraperitoneal injection (IP).

## Methods

### Ethical statement

All procedures involving the use of animals were performed in accordance with a CDC Institutional Animal Care and Use Committee-approved protocol.

### Bacterial strains and growth conditions

*C. burnetii* strains and culture conditions were as previously described [11].

### Murine infections

Murine infections were carried out as previously described, with the following changes [11]. Mice (*n* = 5 per group, unless otherwise stated) were infected by either OG, IP, or AC to deliver a range of doses given in four inoculums suspended in phosphate-buffered saline (PBS) (Table 1). Mice inoculated by AC were dosed using the Biaera aerosol management platform (AeroMP, Biaera Technologies) as previously described [14]. Negative control mice (*n* = 2) were given sterile PBS by each respective routes. Mice were housed in HEPA-filtered isolator cages and separated by infectious route, strain, and dose. On days 7 and 14 post-infection (pi), blood samples were collected from the submandibular vein using 3 mm Goldenrod Animal Lancets (Fisher Scientific) and Microvette 100 serum collection tubes (20.1280.100, Sarstedt). Mice were euthanized on day 21 and blood collected by cardiac puncture. Lungs, spleen, liver, mesenteric lymph nodes (MLN), stomach, kidneys, and heart were aseptically removed and processed [11]. Cohorts infected with NMI at 10^3^ by IP or with CM-SC1 at 10^6^ by IP and OG contained 4 mice per group.

**Table 1.** Dosage chart of *C. burnetii* isolates and infection routes

Target dose		1 × 10^**8**^	1 × 10^**7**^	1 × 10^**6**^	1 × 10^**5**^	1 × 10^**4**^	1 × 10^**3**^	1 × 10^**2**^
Strain	Route	Actual dose received* (cohort size)						
NMI	AC^Ϯ^			1.40 × 10^6^ (*n* = 5)	1.65 × 10^5^ (*n* = 5)	1.08 × 10^4^ (*n* = 5)	7.81 × 10^2^ (*n* = 5)	
	OG	1.15 × 10^8^ (*n* = 5)	1.65 × 10^7^ (*n* = 5)	1.10 × 10^6^ (*n* = 5)	9.20 × 10^4^ (*n* = 5)			
	IP				9.20 × 10^4^ (*n* = 5)	1.90 × 10^4^ (*n* = 5)	1.45 × 10^3^ (*n* = 4)	2.50 × 10^2^ (*n* = 5)
CM-SC1	AC^Ϯ^				2.34 × 10^4^ (*n* = 5)	3.72 × 10^2^ (*n* = 5)	<LD^#^ (*n* = 5)	<LD^#^ (*n* = 5)
	OG	3.45 × 10^8^ (*n* = 5)	6.50 × 10^7^ (*n* = 5)	1.04 × 10^7^ (*n* = 4)	1.02 × 10^6^ (*n* = 5)			
	IP		6.50 × 10^7^ (*n* = 5)	1.04 × 10^7^ (*n* = 4)	1.02 × 10^6^ (*n* = 5)	1.06 × 10^5^ (*n* = 5)		
GP-CO1	AC^Ϯ^		3.09 × 10^6^ (*n* = 5)	3.61 × 10^5^ (*n* = 5)	3.48 × 10^4^ *(n* = 5)	9.45 × 10^3^ (*n* = 5)		
	OG	2.06 × 10^8^ (*n* = 5)	1.11 × 10^7^ (*n* = 5)	1.45 × 10^6^ (*n* = 5)	1.28 × 10^5^ (*n* = 5)			
	IP	2.06 × 10^8^ (*n* = 5)	1.11 × 10^7^ (*n* = 5)	1.45 × 10^6^ (*n* = 5)	1.28 × 10^5^ (*n* = 5)			

*Inoculums were generated from frozen stocks and quantified by *com1* PCR.

^Ϯ^Doses are estimated based on *com1* PCR quantification of the impinger sample following aerosolization as described previously [14].

^#^Below the limit of detection.

### Assessing bacterial burden in tissues

*C. burnetii* DNA was extracted from murine tissues as previously described, with the following changes [11]. DNA extractions were performed using the KingFisher Flex System in conjunction with the KingFisher Cell and Tissue DNA Kit (Thermo Fisher Scientific) according to the manufacturer’s instructions. Quantitative PCR was performed and reported as previously described [11].

### Serological analysis

Serological analysis was performed as previously described [11].

### Statistics

Statistical significance of murine body weights and splenomegaly data were determined based on unpaired Students t-test, and *p* < 0.05 was considered significant. For statistical analysis, all PBS controls regardless of route of administration were pooled (*n* = 6).

## Results

### *Infection of mice by oral gavage with the* C. burnetii *NMI strain is dose dependent and inferior to the aerosol and injection routes.*

**Figure 1.**
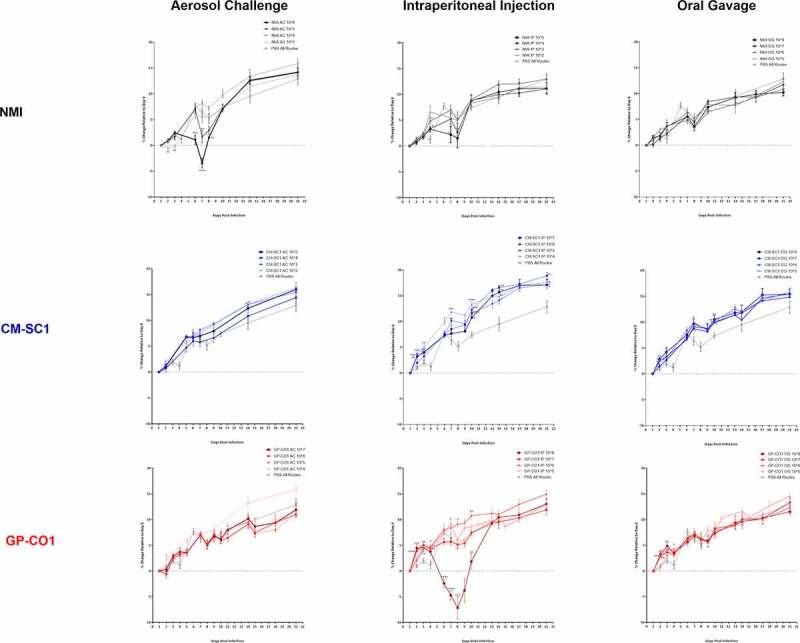
**Body weight following administration of increasing doses of *C. burnetii* in BALB/c mice**. Mice (*n* = 5 unless otherwise stated) were administered either NMI (black), CM-SC1 (blue), or GP-CO1 (red) by aerosol challenge, intraperitoneal injection, or oral gavage. Increasing doses are indicated by increasing pigmentation and size/weight of the symbol/line. Sterile PBS (dashed line/open circle) was delivered via each route as negative controls and data from all three routes are combined (*n* = 6). Displayed are the changes in weight as a percentage relative to day 0 (mean ± SEM). Statistical significance was determined by a Student’s *t*-test relative to PBS controls, *p < 0.05, **p < 0.01, ***p < 0.001, and ****p < 0.0001. No statistical analysis was performed for days lacking PBS control weights. Cohorts infected with NMI at 10^3^ by IP or with CM-SC1 at 10^6^ by IP and OG contained four mice per group

Mice were weighed periodically throughout the 21-day study. Mice infected with NMI *C. burnetii* by AC at a dose of 10^6^ displayed significant weight loss relative to PBS controls by any route (*n* = 6), and by 7 days post infection (pi), they had lost an average of 3.4% (*p* < 0.001) relative to day 1 (Figure 1). No cohorts infected with NMI by IP or OG showed weight loss relative to day 1 including mice receiving the highly concentrated inoculum of 10^8^ by OG. Infections did not result in mortality of any mice regardless of dose or route of administration.

**Figure 2.**
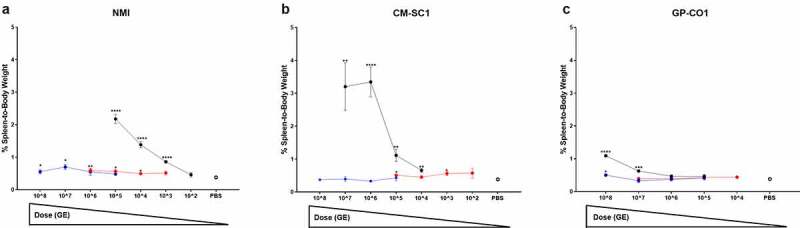
**Splenomegaly following administration of increasing doses of *C. burnetii* in BALB/c mice**. Data is presented as mean spleen-to-body weight ± SEM for cohorts (*n* = 5 unless otherwise stated) administered (a) NMI, (b) CM-SC1, or (c) GP-CO1 by aerosol challenge (red), intraperitoneal injection (black), or oral gavage (blue). Sterile PBS (open circle) was delivered via each route as negative controls and data from all three routes are combined (*n* = 6). Statistical significance was determined by a Student’s *t*-test relative to PBS controls, *p < 0.05, **p < 0.01, ***p < 0.001, and ****p < 0.0001. Cohorts infected with NMI at 10^3^ by IP or with CM-SC1 at 10^6^ by IP and OG contained four mice per group

Splenomegaly was measured at 21 days pi and was highest in mice receiving inoculums via IP (Figure 2). The average spleen-to-body weight for mice receiving PBS by any route (*n* = 6) was 0.38%. The maximum dose of NMI received by IP was 10^5^ and the average spleen-to-body weight was 2.18%, which was statistically significant, *p* < 0.0001 relative to PBS control mice. For mice receiving 10^2^ NMI by IP, splenomegaly was 0.46% and no longer significant (*p* = 0.3). The highest dose of NMI delivered via AC was 10^6^, which induced significant splenomegaly (0.6%, *p* = 0.008). Dosing with NMI by AC at 10^3^ resulted in a spleen-to-body weight that was unremarkable (0.52%, *p* = 0.07). At the highest dose of 10^8^ for NMI OG, splenomegaly was 0.56% (*p* = 0.03), and the lowest dose received by OG at 10^5^ was 0.48% (*p* = 0.02), 1.2- and 4.5-fold lower than by AC or by IP, respectively, at the same dose.

**Figure 3.**
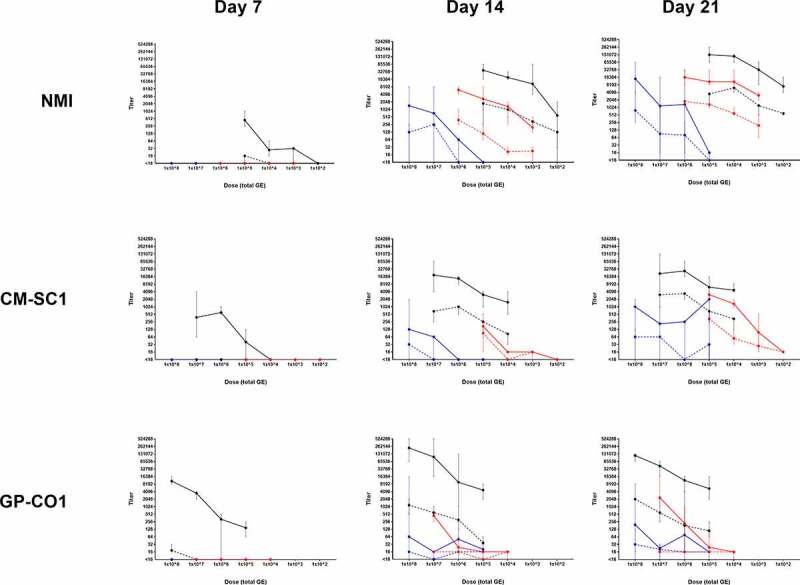
**Serological responses following administration of increasing doses of *C. burnetii* in BALB/c mice**. Serum samples were screened by IFA to determine anti-*C. burnetii* IgG titers against Nine Mile phase I (dashed line) and phase II (solid line) antigens. Data are presented as GMT ± range for cohorts (*n* = 5, unless otherwise stated) receiving NMI, CM-SC1, or GP-CO1 via aerosol challenge (red), intraperitoneal injection (black), or oral gavage (blue). Data points at <16 represent titers below the limit of detection. Cohorts infected with NMI at 10^3^ by IP or with CM-SC1 at 10^6^ by IP and OG contained four mice per group

Development of anti-*C. burnetii* IgG antibodies against phase I and phase II antigen was monitored at days 7, 14, and 21 pi (Figure 3). No evidence of antibody development occurred in PBS controls (data not shown). In all instances of seroconversion, phase II antibody titers were elevated relative to phase I. For mice infected by IP, antibodies had developed by day 7 pi and seroconversion was evident by 14 days pi for all routes; however, some doses were too low to induce a serological response. At 14 days pi, GMT for mice receiving NMI by OG followed a dose-dependent pattern with no seroconversion observed at 10^5^ followed by an increase to 64 (range: <16–64) at 10^6^, 724 (range: <16–8,192) at 10^7^, and 1,448 (range: <16–8,192) at 10^8^. NMI OG-infected mice at 10^5^ continued to have negligible phase II titers of 16 at 21 days pi; however, the phase II GMT reached 14,263 (range: 4,096–65,536) at a dose of 10^8^, increasing from 1,351 (range: 16–32,768) at 10^6^ and 1,176 (range: 16–16,384) at 10^7^. At 10^5^, NMI AC- and IP-infected mice had phase II GMT 10,809 (range: 8,192–32,768) and 131,072 (range: 65,536–262,144), respectively.

**Figure 4.**
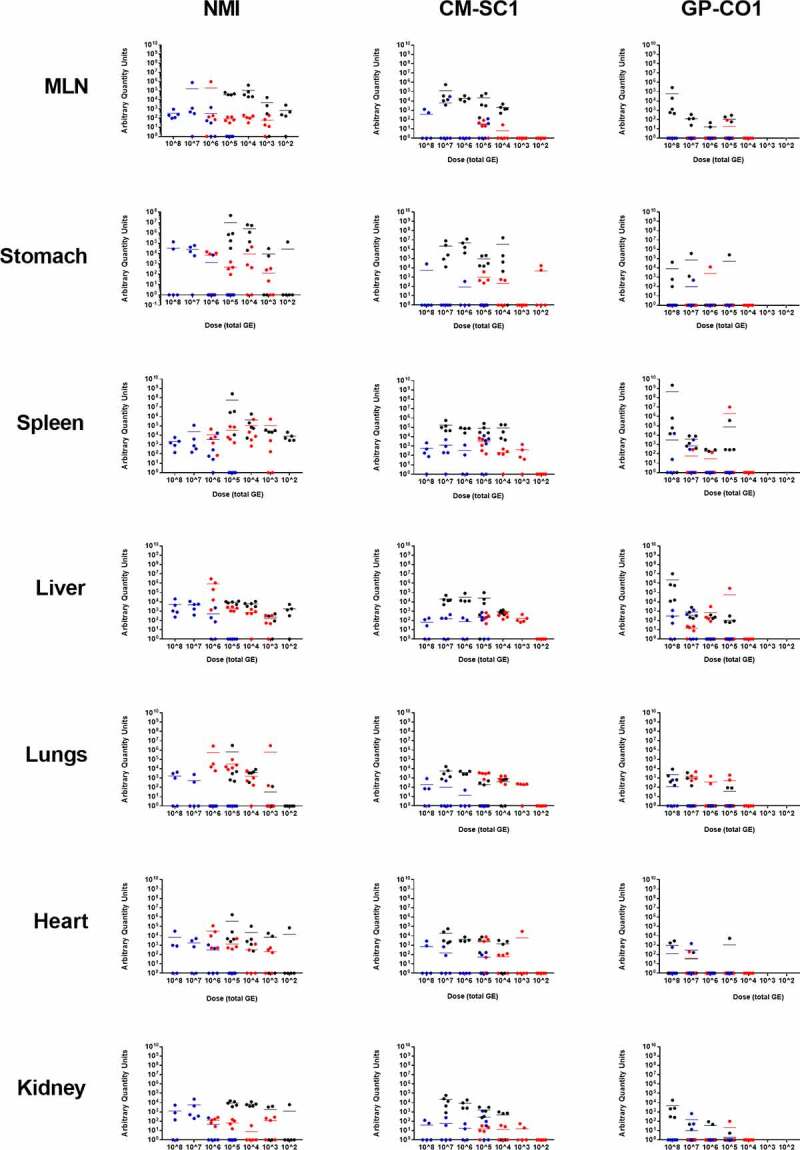
**Bacterial load in tissues following administration of increasing doses of *C. burnetii* in BALB/c mice**. Tissues were analyzed for *C. burnetii* DNA by quantitative PCR. Data is presented as arbitrary quantity units calculated as previously described [21]. Each symbol represents an individual mouse and data include the mean (bar) for cohorts (n = 5, unless otherwise stated) receiving NMI, CM-SC1, or GP-CO1 via aerosol challenge (red), intraperitoneal injection (black), or oral gavage (blue). Symbols at 10° represent values below the limit of detection. Cohorts infected with NMI at 10^3^ by IP or with CM-SC1 at 10^6^ by IP and OG contained four mice per group

At 21 days pi, tissues were analyzed for the presence of *C. burnetii* DNA using quantitative PCR (Figure 4). Tissues from PBS-treated mice were negative (data not shown). In mice treated with NMI by OG, no *C. burnetii* DNA was detectable in any of the tested tissues (MLN, stomach, spleen, liver, lungs, heart, and kidneys) at a dose of 10^5^. In contrast, when the same dose was administered via AC, substantial bacterial loads were detected in all tested tissues. Splenic *C. burnetii* content increased 1,633-fold when introduced via IP relative to AC at 10^5^. Increasing the OG dose to 10^6^ resulted in measurable *C. burnetii* DNA in all tested tissues except the lungs. At 10^7^ for NMI OG-infected mice, bacterial loads had increased across all tested tissues with 17.3- and 7-fold increases for the stomach and spleen, respectively, and could also be detected in the lungs. At the maximum dose of 10^8^, the amount of *C. burnetii* in the tissues plateaued or was reduced demonstrated by fold changes of 1.3 and 11.5 relative to 10^7^ for the stomach and spleen, respectively. At the low dose of 10^3^ for mice challenged with NMI by AC, *C. burnetii* could still be detected in all tissue types tested by 21 days pi. Similarly, the low dose of 10^2^ for mice receiving NMI by IP led to bacterial loads in all tested tissues except the lungs.

### *Infection of mice by oral gavage with the* C. burnetii *contemporary strains, CM-SC1 and GP-CO1, does not follow a dose-dependent pattern and is inferior to the aerosol and injection routes*

The only contemporary strain cohort to lose weight were those receiving 10^8^ GP-CO1 by IP, which resulted in an average loss of 7.2% (*p* < 0.001) by 8 days pi. No weight loss was observed for mice receiving CM-SC1 regardless of the route or dose received. Cohorts receiving CM-SC1 by IP and OG displayed increased weight gain relative to PBS controls (7.38%) with increases by day 10 of 10.8% (*p* = 0.012) and 10.0% (*p* = 0.001) at 10^7^, respectively.

CM-SC1 IP mice had an average spleen-to-body weight of 3.35% (*p* < 0.001) at a dose of 10^6^, which was 10.1-fold higher than for mice receiving the same dose via OG. Splenomegaly remained significant for mice receiving CM-SC1 by IP as low as 10^4^ (0.66%, *p* = 0.003). Cohorts receiving CM-SC1 by AC at 10^5^ and 10^3^ measured significant spleen-to-body weights of 0.51% (*p* = 0.02) and 0.56% (*p* = 0.02), respectively; however, 10^4^ (0.45%) was not significant (*p* = 0.18). Splenomegaly was not observed for AC mice at 10^2^ nor for any mice receiving CM-SC1 by OG regardless of the dose administered.

Mice receiving GP-CO1 by IP at a dose of 10^8^ had an average spleen-to-body weight of 1.09% (*p* < 0.0001), 2.2-fold more than OG at the same dose (0.5%, *p* = 0.03). At 10^7^, GP-CO1 IP mice had an average of 0.63% (*p* < 0.001), 1.6- and 1.9-fold higher than those infected via AC (0.4%, *p* = 0.76) and OG (0.33%, *p* = 0.42), respectively. Spleen-to-body weights for mice dosed at 10^6^ or lower were unremarkable regardless of the administration route.

Seropositivity following CM-SC1 OG infection did not appear to follow an inoculum-dependent pattern at day 21 pi with a phase II GMT of 1,025 (range: <16–2,048) at 10^8^, 215 (range: <16–1,024) at 10^7^, 256 (range <16–256) at 10^6^, and 2,048 (range: <16–2,048) at 10^5^. At a dose of 10^5^, the phase II GMT was 3,104 (range: 2,048–4,096) and 6,208 (range: 2,048–16,384) for AC and IP, respectively. Similarly, GP-CO1 OG-infected mice displayed variable phase II GMTs regardless of the dose received with phase II GMTs of 194 (range: 16–8,192) at 10^8^, 23 (range: <16–32) at 10^7^, 76 (range: <16–4,096) at 10^6^, and 16 (range: <16–16) at 10^5^. At 10^7^, the phase II GMT was 2,353 (range: 1,024–16,384) and 43,238 (range: 16,384–65,536) for GP-CO1 AC and IP, respectively.

Mice infected with CM-SC1 by OG had variable levels of *C. burnetii* in the tissues regardless of the dose administered as evidenced by the 7-, 3-, and 12-fold decreases between loads in the spleen at 10^8^, 10^7^, and 10^6^, respectively, relative to 10^5^. Similarly, mice infected with CM-SC1 by IP displayed stagnant levels of *C. burnetii* in the spleen as the dose increased from 10^4^ to 10^7^. When administered at 10^2^ via AC, CM-SC1 could not be detected in any tissues except the stomach. Increasing the dose to 10^3^ led to detectable levels of CM-SC1 in the spleen, liver, lungs, heart, and kidneys. At 10^5^, the quantity of CM-SC1 in the spleen was 29- and 20-fold higher when administered via IP relative to AC and OG, respectively. Although average loads of CM-SC1 in the spleens were comparable between AC- and OG-infected mice at 10^5^, AC-infected mice had detectable *C. burnetii* in the spleens of all mice, whereas CM-SC1 was only detected in three of the five mice tested when the same dose was delivered via OG.

Infection with the GP-CO1 strain led to sporadic dissemination to peripheral tissues by day 21 pi, despite the route of administration or dose received. At 10^8^ by OG, only two of five mice had detectable levels of *C. burnetii* in the spleen, while at the same dose by IP, four of the five mice had evidence of dissemination to the spleen. Only one of the five mice had GP-CO1 DNA in the spleen at 10^7^ when infected via AC. At 10^7^ by IP, loads in the spleen were 5-fold higher than OG, while AC was 12-fold lower than OG. The difference between OG and IP in the spleen at 10^8^ jumped to 148,000-fold, with similar patterns observed in the liver and kidneys.

## Discussion

Through this work, we have demonstrated the pathology of *C. burnetii* infection in mice resulting from variable doses of three unique isolates administered by three different routes. A high dose (10^8^) of the historic laboratory strain, NMI, delivered directly into the stomach of immunocompetent BALB/c mice produced splenomegaly, a robust antibody response, and colonization of all tissues tested. Conversely, a low dose (10^5^) of NMI by OG was insufficient to cause disease in any of the mice as evidenced by the lack of splenomegaly, seroconversion, and dissemination. Introduction of NMI by IP at a dose 1,000-fold lower or by AC at a dose 100-fold lower was sufficient to induce a robust immune response and lead to dissemination. Our results are in line with what has been described previously by Durand *et al*., who demonstrated a 10,000-fold difference between the infectious dose of IP versus oral infection [15]. Despite being able to cause disease in a dose-dependent manner in immunocompetent mice, the introduction of *C. burnetii* via the oral route is the least efficient infectious route tested herein.

**Figure 5.**
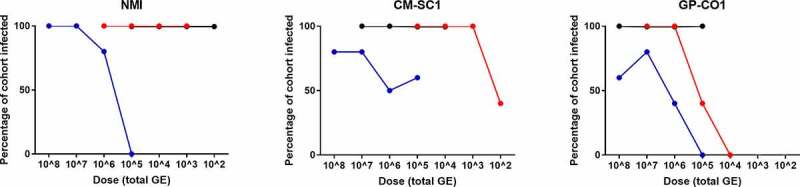
**Percentage of cohorts with any evidence of infection following administration of increasing doses of *C. burnetii* in BALB/c mice**. Displayed are the percentages of mice within each cohort with any evidence of infection based on a serological titer against either phase I or phase II of ≥ 32 or *C. burnetii* detected in at least one of the tissues tested. Percentages (*n* = 5, unless otherwise stated) are shown for mice receiving either NMI, CM-SC1, or GP-CO1 via aerosol challenge (red), intraperitoneal injection (black), or oral gavage (blue). Cohorts infected with NMI at 10^3^ by IP or with CM-SC1 at 10^6^ by IP and OG contained four mice per group

Not only were the immune responses and bacterial loads diminished in mice administered *C. burnetii* via the oral route, but infection success was also inferior to the other more conventional routes as demonstrated in Figure 5. While 100% of mice administered high dose (10^8^) NMI demonstrated signs of infection (titer ≥32 or detectible *C. burnetii* in any tested tissue), a lower dose (10^6^) resulted in infection of 100% of the AC cohort compared to 80% of the OG group. Interestingly, the inferior infection success of ingestion is magnified in the low passage contemporary isolates, CM-SC1 (ST20 isolate from raw cow milk) and GP-CO1 (ST8 goat placenta isolate), which have been shown to have decreased pathogenicity relative to the historic laboratory strain NMI [11]. Ingestion at high doses (10^8^) was not sufficient to ensure transmission to 100% of the tested population for either contemporary isolate. Given that these sequence types predominate in the United States ruminant populations, this observed reduction in transmission success may contribute to the relatively small number of Q fever cases attributed to the oral route of infection [16].

ST8 isolates comprise one of the three major sequence types currently circulating in the United States; they are closely associated with goats and are responsible for multiple outbreaks of Q fever across the US [17–19]. Following an ST8-associated outbreak of Q fever in Washington in 2011, *C. burnetii* infection was evident in all 17 goat herds tested across Washington, Montana, and Oregon; however, only 21 cases of human infection were identified and of those only 15 were symptomatic [18]. Similarly, an ST8-associated Q fever outbreak in Colorado, from which the GP-CO1 strain was isolated, resulted in seroconversion of 11 of 138 persons living within 1 mile of the operation [17]. Despite goat abortions occurring in both outbreaks, which allow for the introduction of large amounts of *C. burnetii* into the environment, the scale of these ST8 outbreaks is fairly unremarkable relative to some other recorded Q fever events, and infections are fewer than anticipated, given an infectious dose of 1–10 organisms [2]. Interestingly, we show here that none of the mice showed any evidence of infection by day 21 pi following inhalation of 10,000 GP-CO1 (ST8) organisms, while aerosol challenge of NMI (ST16) at a dose of 1,000 was sufficient to infect 100% of the mice. These findings suggest that the infectious dose of the ST8 isolates is potentially much higher than that of the classic 1–10 organisms touted for the NMI strain. It is important to consider the potential impact that the introduction of a more virulent strain into the US ruminant populations may have, underscoring the need for continued surveillance.

Administration of *C. burnetii* by ingestion, inhalation, and injection led to dissemination in all tested tissue types, including the stomach. Given that mice are infected following aerosolization of *C. burnetii* in a whole-body chamber, it is possible that introduction of *C. burnetii* into the stomach resulted from grooming; however, dissemination to the stomach from the lungs cannot be ruled out. Interestingly, a study examining the spread of radiolabeled *Francisella tularensis* following inhalation found dissemination to the stomach and other GI tract tissues within hours despite several modifications to eliminate the potential for accidental introduction of the inoculum to the GI tract [20]. We have demonstrated previously that *C. burnetii* is able to persist for at least 21 days in the stomachs of mice following oral gavage. Therefore, we hypothesized that if *C. burnetii* can disseminate to the stomach during natural infection, the stomach may serve as a location to seed recrudescence leading to development of chronic Q fever. To begin to address this, we examined a small cohort of mice (*n* = 3) for the potential to persist in the stomach following OG at 31- and 42-days pi. While NMI was still detected in the spleen of some mice and the GMT increased through day 42, *C. burnetii* was below the limit of detection in the stomach of all mice at days 31 and 42 pi (Supplemental Figure 1). Based on these findings, it is unlikely that the stomach harbors *C. burnetii* long term to facilitate recurrence of chronic infection.

Collectively, our findings show that although *C. burnetii* can establish infection in mice following administration by ingestion, inhalation, and injection, infection severity and transmission success are route dependent, with ingestion being the most inefficient.

## Supplementary Material

Supplemental Material

## Data Availability

The authors confirm that the data supporting the findings of this study are available within the article.

## References

[1] Doron S, Gorbach SL. Bacterial Infections: Overview. In: Heggenhougen HK, editor. International Encyclopedia of Public Health. Oxford: Acadmic Press; 2008. p. 273–282.

[2] Miller HK, Priestley RA, Kersh GJ. Q fever: a troubling disease and a challenging diagnosis. Clin Microbiol Newsl. 2021;43(13):109–118. 10.1016/j.clinmicnews.2021.06.003 PMC10493821 37701818

[3] Ghigo E, Colombo MI, Heinzen RA. The *Coxiella burnetii* parasitophorous vacuole. Adv Exp Med Biol. 2012;984:141–169. 22711631 10.1007/978-94-007-4315-1_8

[4] Anderson A, Bijlmer H, Fournier PE, *et al*. Diagnosis and management of Q fever–United States, 2013: recommendations from CDC and the Q fever working group. MMWR Recomm Rep. 2013;62:1–30. 23535757

[5] Schimmer B, Ter Schegget R, Wegdam M, *et al*. The use of a geographic information system to identify a dairy goat farm as the most likely source of an urban Q-fever outbreak. BMC Infect Dis. 2010;10(1):69. 20230650 10.1186/1471-2334-10-69 PMC2848044

[6] Hawker JI, Ayres JG, Blair I, *et al*. A large outbreak of Q fever in the West Midlands: windborne spread into a metropolitan area? Commun Dis Public Health. 1998;1:180–187. 9782633

[7] Enright JB, Sadler WW, Thomas RC. Pasteurization of milk containing the organism of Q fever. Am J Public Health Nations Health. 1957;47(6):695–700. 13424814 10.2105/ajph.47.6.695 PMC1551060

[8] Loftis AD, Priestley RA, Massung RF. Detection of *Coxiella burnetii* in commercially available raw milk from the United States. Foodborne Pathog Dis. 2010;7(12):1453–1456. 20704507 10.1089/fpd.2010.0579

[9] Shah SY, Kovacs C, Tan CD, *et al*. Delayed diagnosis of Q fever endocarditis in a rheumatoid arthritis patient. IDCases. 2015;2(4):94–96. 10.1016/j.idcr.2015.09.002 PMC4712205 26793469

[10] Signs KA, Stobierski MG, Gandhi TN. Q fever cluster among raw milk drinkers in Michigan, 2011. Clin Infect Dis. 2012;55(1387–1389):1387–1389. 10.1093/cid/cis690 22893578

[11] Miller HK, Priestley RA, Kersh GJ. Transmission of *Coxiella burnetii* by ingestion in mice. Epidemiol Infect. 2020;148:e21. 32019625 10.1017/S0950268820000059 PMC7026900

[12] Kruszewska D, Tylewska-Wierzbanowska SK. *Coxiella burnetii* penetration into the reproductive system of male mice, promoting sexual transmission of infection. Infect Immun. 1993;61:4188-4195. 10.1128/iai.61.10.4188-4195.1993 PMC281143 8406807

[13] Krumbiegel ER, Wisniewski HJ. Q fever in the Milwaukee area. II. Consumption of infected raw milk by human volunteers. Arch Environ Health. 1970;21(1):63–65. 5467889 10.1080/00039896.1970.10667193

[14] Schoffelen T, Self JS, Fitzpatrick KA, *et al*. Early cytokine and antibody responses against *Coxiella burnetii* in aerosol infection of BALB/c mice. Diagn Microbiol Infect Dis. 2015;81(4):234–239. 25618420 10.1016/j.diagmicrobio.2014.12.008 PMC4740919

[15] Durand MP. [Lacteal and placental excretion of *Coxiella burnetii*, agent of Q fever, in the cow. Importance and prevention]. Bull Acad Natl Med. 1993;177:935–945. discussion 945-936. 8221191

[16] Gale P, Kelly L, Mearns R, et al. Q fever through consumption of unpasteurised milk and milk products - a risk profile and exposure assessment. J Appl Microbiol. 2015;118(5):1083–1095. 25692216 10.1111/jam.12778

[17] Bamberg WM, Pape WJ, Beebe JL, *et al*. Outbreak of Q fever associated with a horse-boarding ranch, Colorado, 2005. Vector Borne Zoonotic Dis. 2007;7(3):394–402. 10.1089/vbz.2007.0104 17896873

[18] Bjork A, Marsden-Haug N, Nett RJ, *et al*. First reported multistate human Q fever outbreak in the United States, 2011. Vector Borne Zoonotic Dis. 2014;14(10):111–117. 10.1089/vbz.2012.1202 24350648

[19] Kersh GJ, Priestley RA, Hornstra HM, *et al*. Genotyping and axenic growth of *Coxiella burnetii* isolates found in the United States environment. Vector Borne Zoonotic Dis. 2016;16(9):588–594. 27304166 10.1089/vbz.2016.1972 PMC5011622

[20] Ojeda SS, Wang ZI, Mares CA, *et al*. Rapid dissemination of *Francisella tularensis* and the effect of route of infection. BMC Microbiol. 2008;8(1):215. 19068128 10.1186/1471-2180-8-215 PMC2651876

[21] Kersh GJ, Lambourn DM, Self JS, *et al*. *Coxiella burnetii* infection of a steller sea lion (Eumetopias jubatus) found in Washington State. J Clin Microbiol. 2010;48(9):3428–3431. 20592144 10.1128/JCM.00758-10 PMC2937693

