# Supplementary material for: Comparison of three Coxiella burnetii infectious routes in mice
Source: Virulence. 2021 Sep 27;12(1):2562–70. doi: 10.1080/21505594.2021.1980179 (PMC8477946; doi:10.1080/21505594.2021.1980179)
Supplement: Supplemental Material [file KVIR_A_1980179_SM9675.zip › supplementary/Supplemental Figure legend.docx]

## **Supplemental Figure 1. Bacterial load in tissues and serological responses following administration of NMI *C. burnetii* in BALB/c mice.** NMI at a dose of 10^6^ was introduced via OG (n=3) and **(A)** tissues were assayed for *C. burnetii* DNA by quantitative PCR at days 31- and 42- pi. Data is presented as arbitrary quantity units as previously described^21^. Each symbol represents an individual mouse for bacterial loads in the spleen (black) and stomach (red). Symbols at 10^0^ represent values below the limit of detection. No *C. burnetii* DNA was detected in the MLN, kidneys, liver, lungs, and heart for any mice at either time point (data not shown). **(B)** Serum samples were screened by IFA to determine anti-*C. burnetii* IgG titers against Nine Mile phase I (open symbol/dashed line) and phase II (solid symbol/solid line) antigens. Data are presented as GMT ± range. Data presented for days 7-21 pi are replicated from Figure 3.
