# Supplementary figures and images for: Comparison of three Coxiella burnetii infectious routes in mice
Source: Virulence. 2021 Sep 27;12(1):2562–70. doi: 10.1080/21505594.2021.1980179 (PMC8477946; doi:10.1080/21505594.2021.1980179)

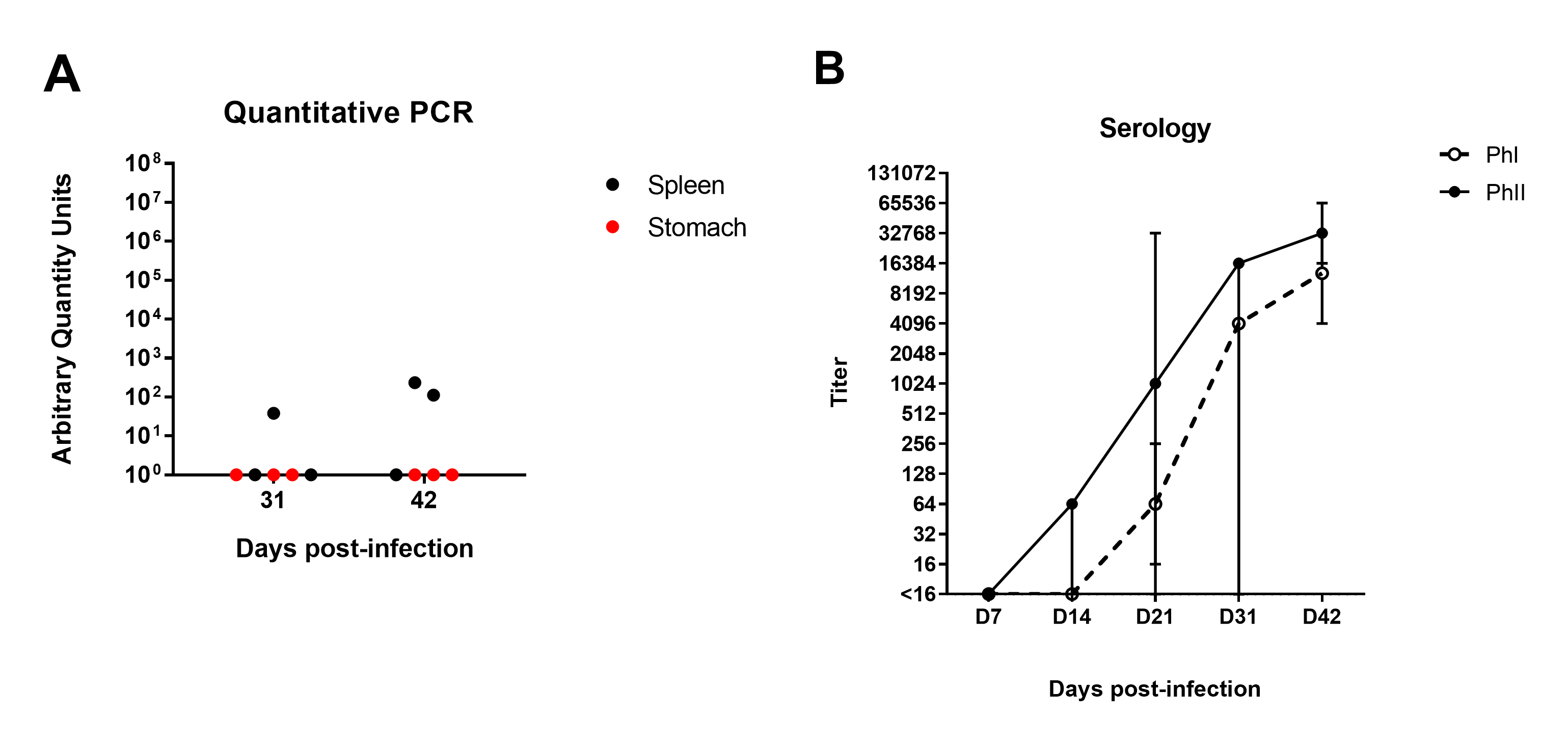

Supplement: Supplemental Material [file KVIR_A_1980179_SM9675.zip › supplementary/Supplemental Figure 1.tif]
